# The role of Leucocyte-rich and platelet-rich fibrin (L-PRF) in the treatment of the medication-related osteonecrosis of the jaws (MRONJ)

**DOI:** 10.4317/jced.54154

**Published:** 2017-08-01

**Authors:** Jorge A. Cano-Durán, Juan-Francisco Peña-Cardelles, Daniel Ortega-Concepción, Víctor M. Paredes-Rodríguez, Mariano García-Riart, Juan López-Quiles

**Affiliations:** 1 Department of Oral Medicine and Surgery, School of Dentistry, Complutense University, Madrid, Spain

## Abstract

**Background:**

For the treatment of the bisphosphonates and other drugs related osteonecrosis of the jaws, currently medication-related osteonecrosis of the jaws (MRONJ), have been established different conservative therapeutic approaches, avoiding surgery except in cases of extreme need. Given the controversy and lack of current consensus regarding MRONJ therapy in patients, new techniques have been developed among which the use of fibrin membranes rich in platelets and leukocytes (L-PRF). The objective of this review is to evaluate whether L-PRF treatment is really effective, as well as the results that can be achieved by this therapeutic alternative.

**Material and Methods:**

A review of the literature in the PubMed/Medline database of all those studies using L-PRF in the treatment of osteonecrosis using the keywords “Osteonecrosis”, “Jaws”, “L-PRF” and “ Leucocyte-rich platelet-rich fibrin “.

**Results:**

The use of L-PRF for the treatment of MRONJ is really effective, especially when it is performed with a simultaneous application of L-PRF and morphogenetic protein-2 (BMP-2), even in patients submitted for long periods of time to therapy with intravenous bisphosphonates. However, success will depend on several factors such as the previous existence of infection or the clinical stage in which the patient is.

**Conclusions:**

The current literature demonstrates the effectiveness of the use of L-PRF in osteonecrosis, and it can be considered as a real alternative in the treatment of this entity. However, more clinical studies are needed to really assess this new therapy.

** Key words:**Osteonecrosis, Jaws, L-PRF, Leucocyte-rich platelet-rich fibrin.

## Introduction

**Figure 1:**
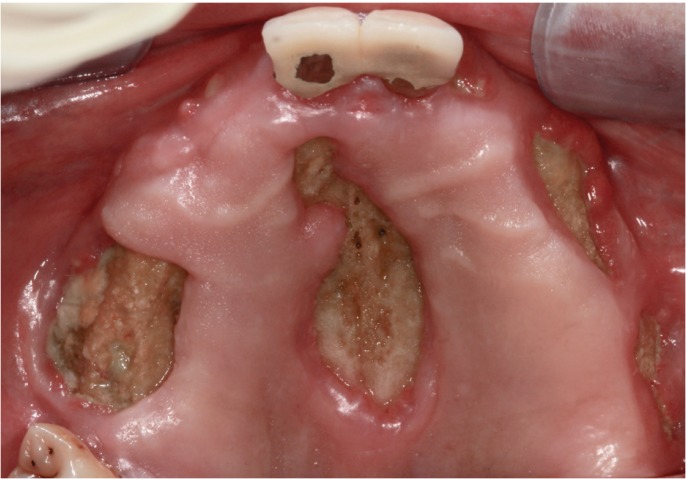
Maxillary osteonecrosis in a patient with prostate cancer and bone metastasis treated with Zometa™ (Zoledronic Acid).

The first evidence in the literature about osteonecrosis of the jaws (ONJ) derived from a treatment with bisphosphonates (BPs) was in charge of Marx in 2003. Since then, documented cases have been taking place in recent years considering the pathology as a major complication as a consequence of the use of this family of drugs (Fig. 1) (1-3).

BPs are stable analogues of pyrophosphate that modulate bone metabolism and that are used in the treatment of bone resorption diseases such as Paget’s disease, osteoporosis, or hypercalcemia associated with different malignant processes such as multiple myelomas, or bone metastases. The mechanism of action of bisphosphonates is based on the inhibition of bone resorption by limiting osteoclastic activity, also exerting an antiangiogenic effect, and therefore they are also used as antitumor therapy (4).

ONJ is defined as an area of necrotic bone that does not heal in the 8-week period in patients who have received treatment with BPs without a history of radiotherapy in the region of the maxilla (5).  Table 1 shows the current classification of this pathology established by the American Association of Oral and Maxillofacial Surgeons (AAOMS) in 2014 (6).

**Table 1:**
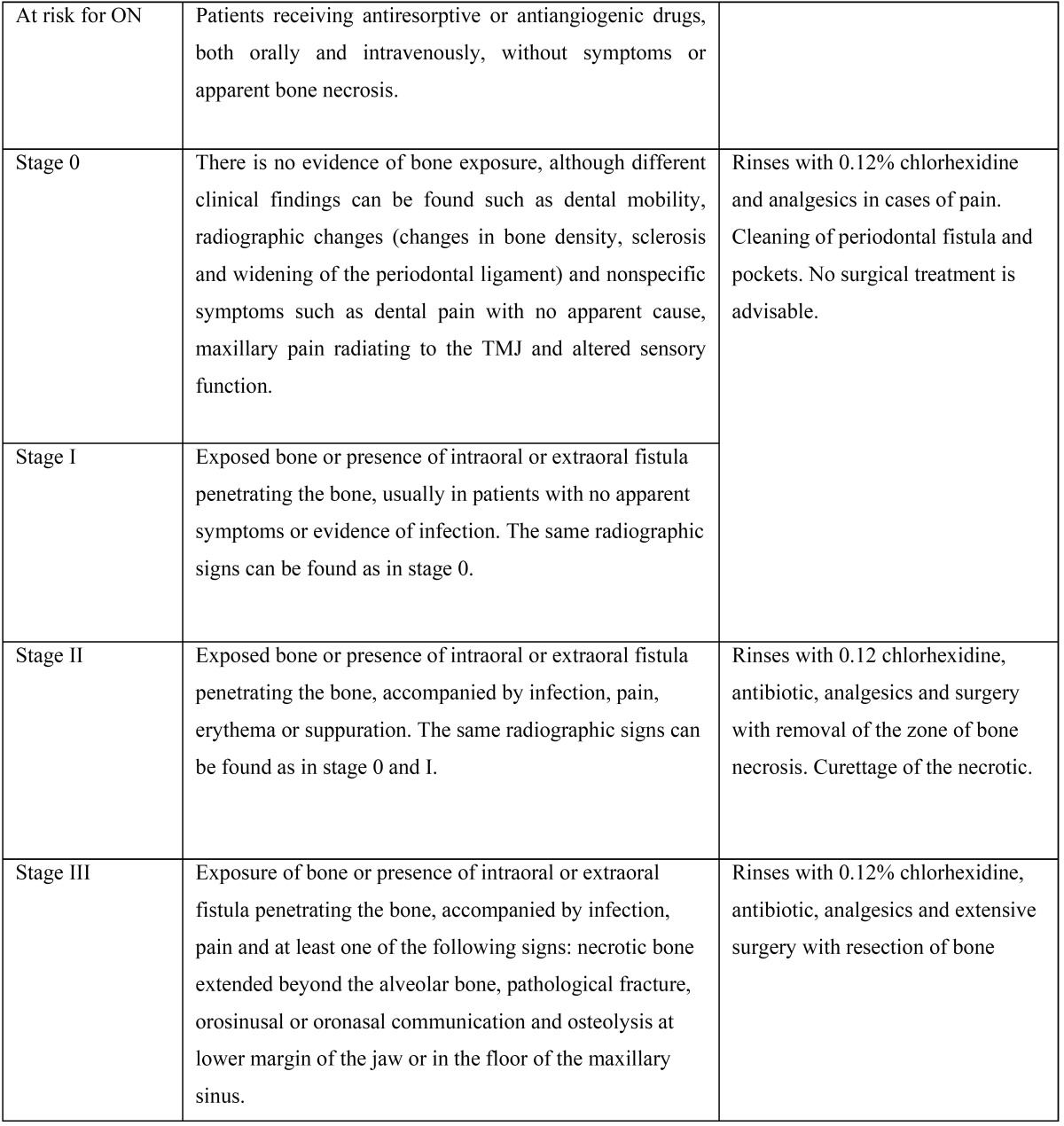
Updated classification of osteonecrosis of the jaws (AAOMS, 2014) (6) and treatment according to the clinical stage (10).

Furthermore, this complication has been associated with the use of other drugs such as Denosumab, an antiresorptive drug of the monoclonal antibody family, used in the same way for the treatment of osteoporosis and for the metastatic bone disease (3). However, this drug has a different mechanism of action than BPs. Meanwhile the latter ones act against active mature osteoclasts, Denosumab acts on the precursors of osteoclasts by inhibiting RANKL, preventing the formation, differentiation and function of osteoclast cells, as well as the associated bone resorption. Because of this, the term bisphosphonate-related osteonecrosis of the jaws (BRONJ) has now been replaced by medication-related osteonecrosis of the jaws (MRONJ) (7,8).

The most recent literature has studied the global incidence of ONJ by BPs and Denosumab, finding very similar results with a percentage between 1-2% (9).

Organizations such as the AAOMS and the American Society for Bone and Mineral Research (ASBMR) have recommended conservative approaches to the treatment of MRONJ. These approaches include the use of antibiotics, oral antimicrobial mouth-washes and minimal debridement of lesions if necessary (10). However, surgical therapy is a controversial attitude today, since it is not always effective and results in an invasive method that in some cases due to trauma on the treated area, may aggravate the necrotic condition (1). Because of this, new therapeutic alternatives have emerged, such as the use of the hormone teriparatide, laser therapy, the use of hyperbaric oxygen, ozone therapy and platelet concentrates, being this last technique one of the newest and promising treatments for the management of MRONJ ( Table 1) (1,11-14).

Platelet concentrates are autologous products, since they are obtained from the individual himself, which contain high concentrations of different growth factors, such as transforming growth factor, platelet-derived growth factor, vascular endothelial growth factor and factor of endothelial growth, all of them secreted by platelets. These concentrates stimulate and accelerate the healing and the bone and tissue regeneration and are therefore used in many fields of medicine. Without going further, several reports have shown promising results for these compounds as a treatment of MRONJ (13,15).

However, a new method was introduced in 2006 that incorporates leukocyte concentrate, which is known as leukocyte and platelet rich fibrin concentrate (L-PRF). It is a physiological material that allows the release of growth factors over a prolonged time, resulting in an acceleration in healing, reducing the risk of contamination, edema and postoperative pain. It is also a completely harmless method, since it is prepared from the patient’s own blood, eliminating the possibility of transmission of parenteral diseases, as well as allergies or rejection immune reactions. From the surgical point of view, it helps in homeostasis, prevents gingival dehiscence and favors the remodeling and healing of both soft and hard tissues (16). All these characteristics have allowed that in 2014, Dinca *et al.* (17) started using L-PRF membranes in the treatment of stage II MRONJ after intravenous bisphosphonate therapy.

**Figure 2:**
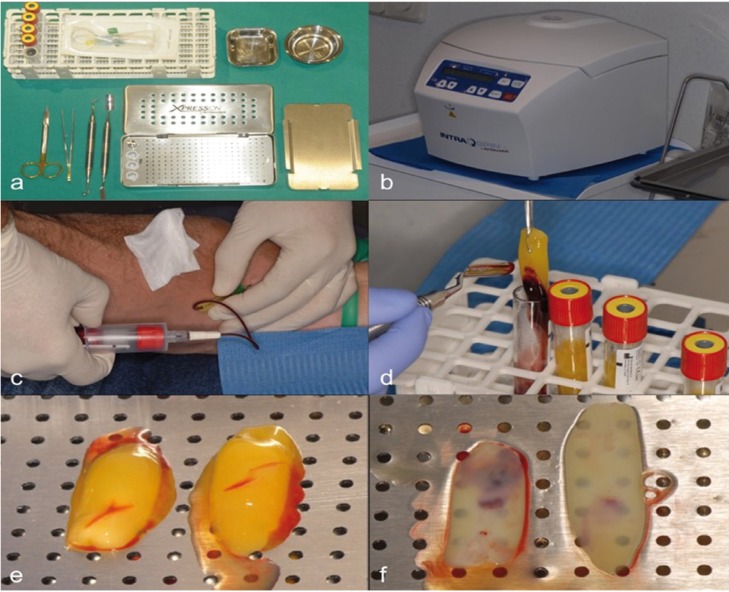
Preparation of L-PRF membrane from the patient’s own blood extraction. (A) Required Kit for the process. (B) Centrifuge used in the process. (C) Blood extraction. (D) Result after blood´s centrifugation. (E) Clot obtained after separation of the fibrin clot. (F) Membrane obtained after clot´s dehydration.

Their obtaining technique consists on the extraction of 10ml of blood from the antecubital vein of the patient and its immediate centrifugation at 3,000 rpm during 10 min or 2,700 rpm during 12 min, producing the blood clotting immediately coming into contact with the walls of the tube. A fibrin membrane is obtained through each blood collection tube. Fibrinogen is initially concentrated in the mid-upper part of the sample tube and, subsequently, circulating thrombin transforms it into fibrin with centrifugation, resulting in the creation of a clot that is located in the middle of the tube. In the lower part are the erythrocytes and, at the top, the acellular plasma. The collected sample corresponds to the clot of fibrin and platelets after separation of the erythrocytes rich layer. For use in osteonecrosis of the jawbone, the resulting clot must be dehydrated, compressing it between two sterile gauze soaked in saline solution or by a specific instrument, thus obtaining a manageable membrane that can be placed directly on the lesion (Fig. 2).

Recently, studies have been conducted on the use of bone morphogenetic protein-2 (BMP-2) in the treatment of osteonecrosis and its role in bone remodeling. This protein has been widely used for the treatment of bone defects due to its osteoinductive capacity and, therefore, it has been thought that BMP-2 has a potential effect on the reversion of the suppressed bone, thus improving bone remodeling (16).

Based on previous studies, Park et al. (2) proposed the hypothesis that the simultaneous application of L-PRF and morphogenetic protein-2 (BMP-2) stimulate soft tissue healing and bone remodeling, thus contributing to success in the treatment of osteonecrosis.

The aim of this research is to study the therapy by using L-PRF membranes in the osteonecrosis of the jaws and to evaluate the results of this treatment to know the efficacy of this therapeutic attitude.

## Material and Methods

For the accomplishment of this review a question was established in order to answer it with the elaboration of this research. This question is: Is treatment with fibrin membranes rich in platelets and leukocytes effective in patients with symptomatic maxillary osteonecrosis?

An advanced literature search was performed using the PubMed / Medline database, looking for the following keywords: “Osteo-necrosis”, “Jaws”, “L-PRF” and “Leucocyte-rich platelet-rich fibrin”. These terms were searched together using the “AND/OR” binding nexuses as follows: ((((Osteonecrosis) AND Jaws) AND L-PRF) OR (((Osteonecrosis) AND Jaws) AND Leucocyte-rich platelet-rich Fibrin).

In addition, in order to complete and ensure the thoroughness of the review of the literature, an additional manual search was made in the references of the articles that were included in the study to find possible studies that were of interest to the research.

Whit the purpose of carry out the choice of the studies, the following inclusion and exclusion criteria were established:

-Inclusion criteria:

• Articles available in full text, regardless of study period or year of publication.

• Articles published in scientific journals and written in English.

-Exclusion criteria. All studies published in a language other than English, as well as comments and letters to the editor, were excluded.

## Results

After the research, we found three articles that relate these terms, due to the novelty of this therapeutic technique. The articles were reviewed and, after the study of references present in them to be interesting for the elaboration of this research paper, a total of 19 articles were included. From this bibliographic review, the following results could be obtained:

In the first study (1) a total of 34 women were included, whose characteristics are shown in  Table 2, which present ONJ associated to the use of bisphosphonates. These patients were treated with L-PRF. The results of the response to treatment are shown in  Table 2. As it can be observed, in only 2 patients (6%) no response was obtained to the treatment. Both were being treated with Zoledronate and had received chemotherapy. After the intervention, the lesions were similar since their necrotic bone and pain persisted up to four months later, at which time a suppurative discharge was performed. In contrast, treatment was effective in most patients (complete remission in 26 patients [77%] and partial remission in 6 patients [18%]). A significant association (*p*=0.002) was found between the response to treatment and the stage of osteonecrosis, since the more advanced the pathology was, the worse was the response to treatment. In none of the cases there were allergic or immunological reactions to L-PRF.

**Table 2:**
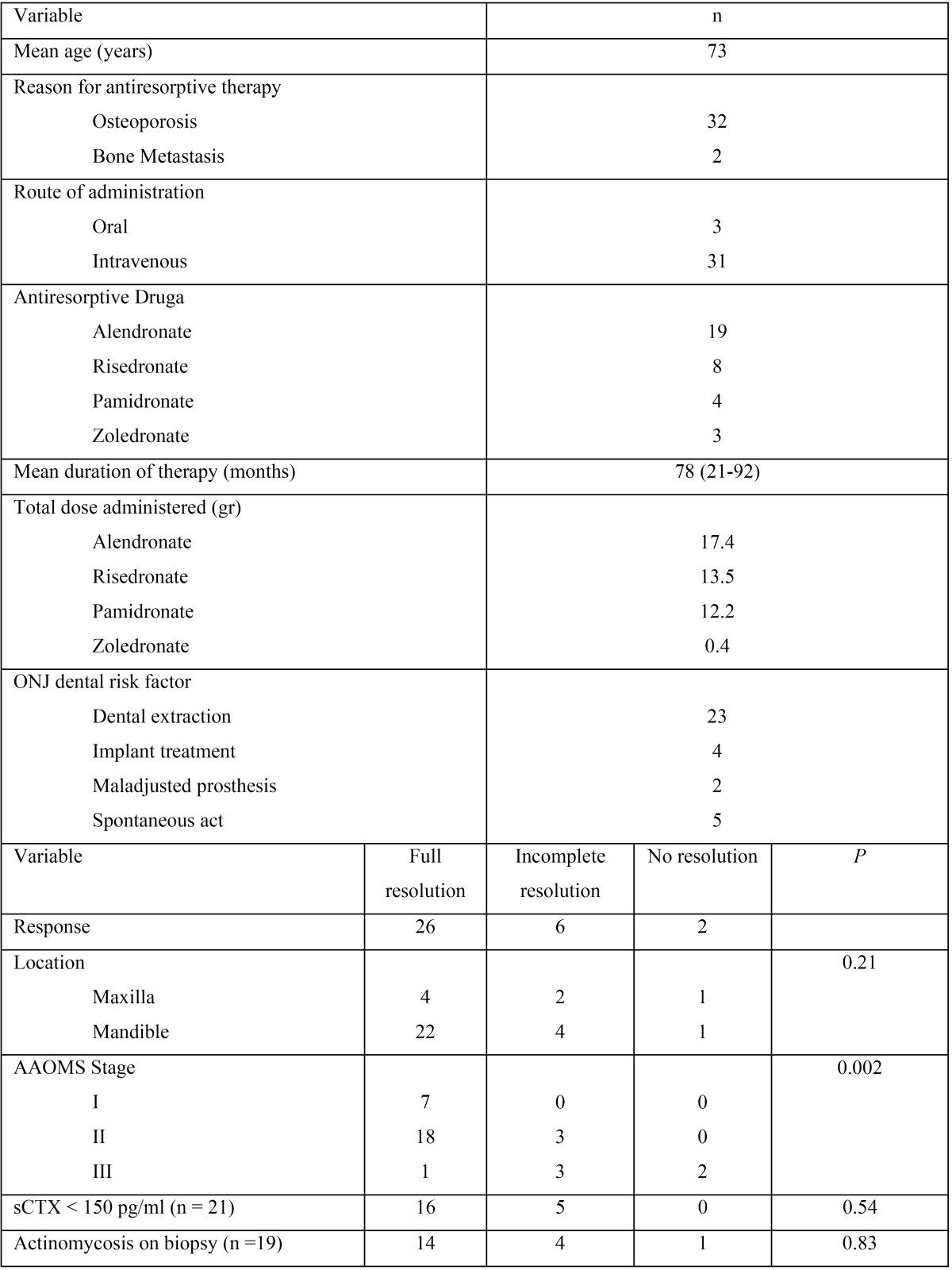
Characteristics of the patients evaluated in the study by Kim JW *et al.* and response to treatment (1).

In the second study (2), was used, a sample of 55 patients, all of them with ONJ. 25 were treated with an application of L-PRF (group 1) and 30 patients were treated with L-PRF and BMP-2 simultaneously (group 2). The characteristics of these patients are shown in  Table 3. On the other hand, the results of the treatment in these two groups are expanded in  Table 4. Moreover, these authors evaluated the association between the resolution of the pathology and the clinical factors of the patients. It was found that the existence of bacterial colonies was a significant factor that negatively affected the resolution of the pathology, because the absence of an active infection at the surgical site led to better healing compared to the non-healing obtained in the patients in which there were signs of infection. There was no significant association between resolution of osteonecrosis and the following clinical factors: sex, age, antiresorptive therapy ratio, route of administration, duration of the antiresorptive therapy, stage, CTX level, steroid use, and medical history of diabetes.

**Table 3:**
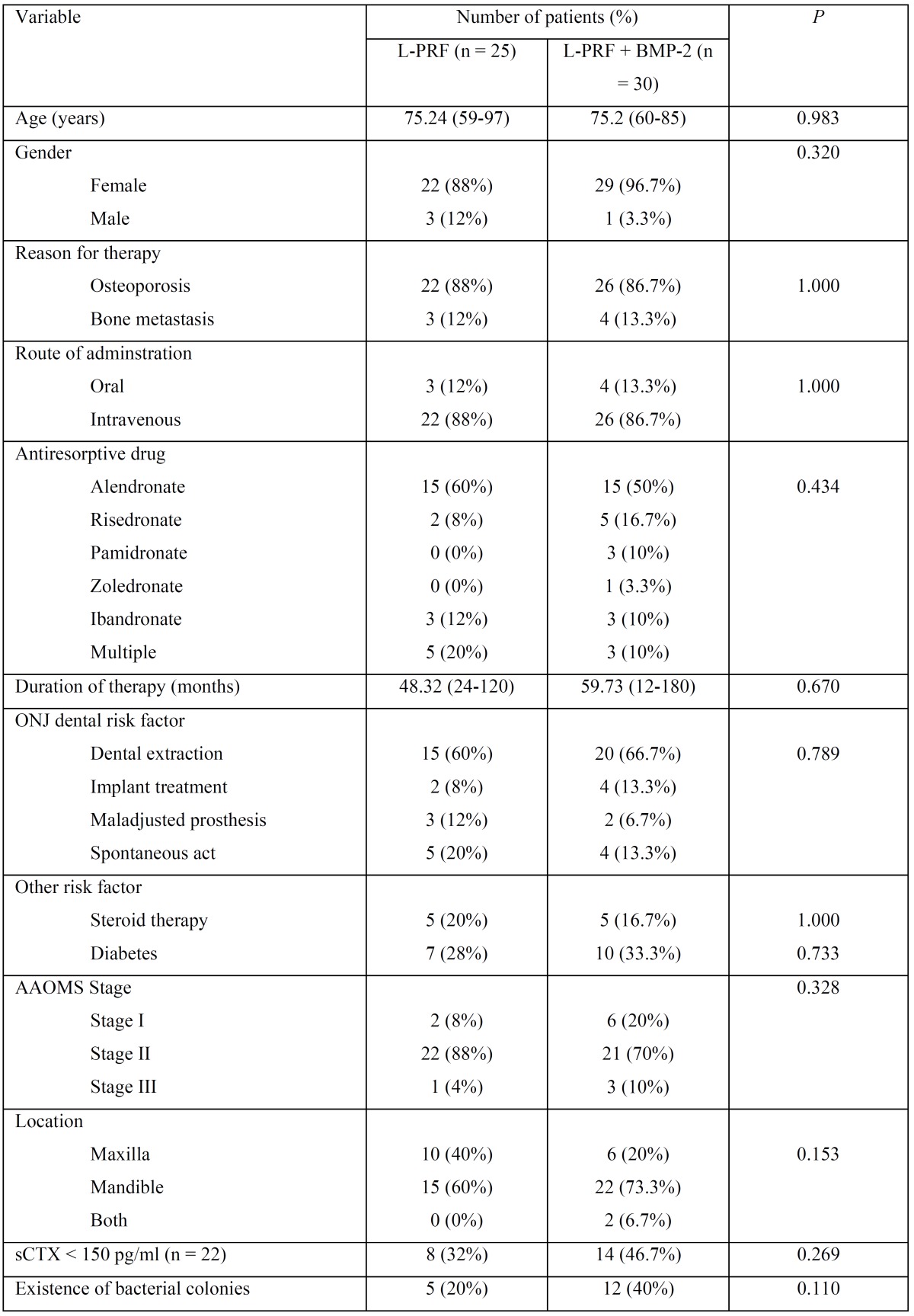
Characteristics of the population studied by Park JH *et al.* (2).

**Table 4:**
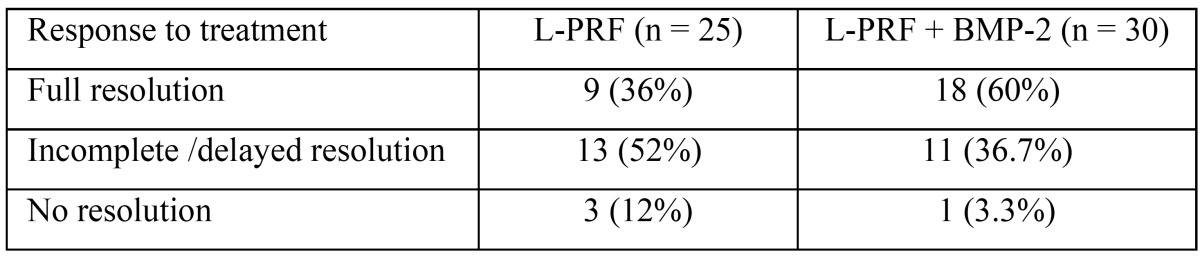
Treatment outcome in the two groups studied by Park *et al.* (2).

In the third study (3) two clinical cases are presented. The first one, refers to a 69-year-old man with lung cancer and bone and liver metastases, medicated with Denosumab and chemotherapy during the last eight months. The second case presents a 44-year-old woman with breast cancer and bone metastases, dosed with Bevacizumab and Denosumab during the last seven months. In both cases, an ONJ in stage II is evaluated after clinical examination, since they refer important pain caused by surgery. In the first case the patient is treated with antibiotic (amoxicillin/clavulanic acid) and surgical therapy combined with L-PRF. Surgery was performed four months after Denosumab discontinuation and, four months later after the treatment, there was a stabilization of the lesion and no symptomatology, so the patient went from stage II to stage I. However, a large, but not complete mucosal coating of the lesion was observed. In the second case, it is also initiated antibiotic therapy (amoxicillin/clavulanic acid) and it is performed a surgery combined with the use of L-PRF membranes after discontinuation of Bevacizumab and Denosumab 30 days before. As in the previous case, the patient went on to stage I and there was a partial overlap of the lesion.

## Discussion

The aim of this research was to evaluate, based on the results obtained in the researches of other authors, whether if L-PRF membranes are an effective and predictable method in the treatment of osteonecrosis, since there is currently no therapy available universally accepted and new less invasive therapeutic alternatives are being looked for that help the healing of the MRONJ.

The study done by Kim *et al.* (1) should be considered of significant importance since it was the first to show promising results in the treatment after the application of L-PRF in a group of patients who presented BRONJ. The disease was diagnosed also by examination and medical history as well as by imaging diagnosis, including computed tomography scan (CT) and bone scan. In addition, these authors evaluated the action of the morphogenetic proteins (BMPs) associated with the matrix of L-PRF by the possibility of contributing to the induction of bone healing and the added leukocytes in the platelet concentrates by their antimicrobial activity, immunological regulation and the ability to produce large amounts of vascular endothelial growth factor, and although more studies are needed to elucidate these functions, they obtained quite positive results.

This study evaluated the response to treatment with L-PRF in function of different characteristics of the osteonecrosis as the site of the injury and the size of exposed necrotic bone, presence of infection (mainly actinomycosis), pain, ONJ stage, type of BPs used, as well as their dose, route of administration, duration of treatment and indication for use. Prior to the application of L-PRF, all patients underwent antibiotic and analgesic treatment and thorough oral hygiene. Furthermore, surgical debridement of all areas of bone with infection was performed, as well as a sequestration and ostectomy until a bleeding bone bed was obtained. After a therapeutic period of between 21-92 months depending on each case, it was possible to evaluate that the conservative approaches with L-PRF are quite effective, but that when the disease is more advanced a more active intervention is necessary to prevent its progress. As for the biomarkers for predicting the risk of osteonecrosis (sCTX24 particularly) and the presence of actinomycosis, there was no significant association between the response to L-PRF and the low concentration of CTX or the presence of actinomycosis, although it must be taken into account that the presence of actinomycosis is associated as an important added risk factor. However, this study presents the great limitation of not having a control group, in addition to the fact that the sample, although sufficient to establish an initial conclusion about this new therapy, is limited, being also not very homogeneous (all subjects were women) unable to establish thus possible differences between sexes.

Another study realized by Park *et al.* (2) attempted to investigate the additional effect of BMP-2 on L-PRF in the treatment of osteonecrosis. This research resulted from the existing controversy over the absence of BMPs within L-PRF, and there is no consensus as to whether it is an effective treatment. It is known that BMP-2 plays an important role in improving bone remodeling and these authors had the hypothesis that associating it with leukocyte rich fibrin and platelet stimulates both soft and hard tissue healing, contributing to the therapeutic success. In this paper, we compared the results of healing of these lesions in two groups of patients: one treated with L-PRF and another with a combination of both compounds. This study demonstrated a significant association between resolution of pathology and combined therapy of L-PRF and BMP-2 compared to the second group. The differences between the two studies were mainly exhibited in the healing periods, because the healing pattern was more accelerated in the group where both components were used synergistically.

Studying the L-PRF matrix, these authors stated that it dissolves slowly, allowing the progressive release of cytokines and plate-let-derived growth factors, acting as an anti-infective agent with a key role in immune regulation. Therefore, it accelerates the healing of epithelial wounds, promotes tissue vascularization and improves soft tissue regeneration. However, considering the absence of BMPs (protein with osteoinductive capacity and therefore able to favor bone healing), they questioned the direct therapeutic effect of L-PRF in the cure of osteonecrosis, since it is mainly a bone disease (2).

According to this review, the most common clinical findings in patients with ONJ are classic signs of infection, with bacterial colonization occurring in more than 80% of patients with this pathology. Actinomyces has been highlighted as the most common microorganism and it has been shown that lesions infected by this bacterium require a longer treatment due to a significantly prolonged duration of disease. In the present study, the active infection of the lesion was a negative result in the resolution of the disease, since the infection prevents healing. Therefore, it was stated that the conservative management with antibiotics is necessary to control the infection and to be able to obtain an effective result in the treatment with L-PRF (2).

Finally, the efficacy of surgical treatment in association with L-PRF and BMP-2 was studied, since despite the opinions that conservative non-surgical treatment is the preferred method, there seems to be a success rate for surgical therapy of 73-100%. In this work, it could be observed that removal of the necrotic bone until the appearance of bleeding in fresh bone is effective in 96.7% of the cases in which the therapy is combined with L-PRF and BMP-2 and in a 88 % when it is associated only with L-PRF. Recently, a prospective study conducted by Lesclous *et al.* (18) showed that surgical management of the pathology improves mucosal healing and positively influences in the clinical outcome compared with the conservative treatment. A systematic review by Rupel *et al.* (19) reported that the average of the healing rates for surgical treatment were 84% for extensive surgeries, 85% for extensive laser assisted surgeries and 75% for conservative surgery, meanwhile the results of non-surgical treatment were lower with healing rates of 36% for antibiotic therapy and 30-52% for antibiotic therapy in combination with low-power laser therapy or hyperbaric oxygen therapy (2).

In the work carried out by Maluf *et al.* (3) was studied the osteonecrosis associated with the use of Denosumab. This drug is a monoclonal antibody that has a therapeutic efficacy similar to BPs, with less side effects than these, although this new drug continues to have the adverse effect of development of osteonecrosis at the level of the jaws, since its mechanism of action produces the blockade of RANKL to RANK, although due to its young age, the cases described in the literature of osteonecrosis of the jawbones are limited. However, in the published cases available in the literature, the most commonly used treatment is the same protocol as for BRONJ. This therapy is based on a surgical step in order to eliminate the necrotic bone combined with a long-term antibiotic therapy. This study was based on the fact that the use of L-PRF in Denosumab osteonecrosis had never been described before. This work was based on several cases in which leukocyte rich fibrin and platelet were used in the treatment of the pathology and, after follow-up, the patients showed a large but incomplete healing of the wounds. The use of conservative treatments such as L-PRF have a success rate around 20-50%, being lower than the success rates of 85% of surgical therapies. However, if the results were considered positive because, when the surgery was associated with the released growth factors, the natural coagulation process was favored, without contributing any new risks as side effects. Therefore, and although further studies are needed to assess the efficacy of this treatment in the pathology induced by Denosumab, the L-PRF associated with surgery can be considered as a suitable therapeutic attitude in the treatment of this entity.

After the study of the most recent literature available in which the treatment of osteonecrosis through the use of L-PRF membranes is evaluated, it can be concluded that the use of this method is effective in many cases and can therefore be considered as a viable therapeutic alternative.

In addition, a successful simultaneous application of L-PRF and BMP-2 contributes effectively to the success of the treatment. Combined treatment leads to the early resolution of ONJ, so patients with this pathology could benefit from this therapy.

Nevertheless, therapeutic success depends on several factors such as the location of the lesion, the size of the lesion or the moment of diagnosis, so that, despite they are quite encouraging results and they open a new path in the treatment of this pathology, more studies are needed to demonstrate the true efficacy of the therapy.

## References

[1] Kim JW, Kim SJ, Kim MR (2014). Leucocyte-rich and platelet-rich fibrin for the treatment of bisphosphonate-related osteonecrosis of the jaw: a prospective feasibility study. Br J Oral Maxillofac Surg.

[2] Park JH, Kim JW, Kim SJ (2017). Does the Addition of Bone Morphogenetic Protein 2 to Platelet-Rich Fibrin Improve Healing After Treatment for Medication-Related Osteonecrosis of the Jaw?. J Oral Maxillofac Surg.

[3] Maluf G, Pinho MC, Cunha SR, Santos PS, Fregnani ER (2016). Surgery Combined with LPRF in Denosumab Osteonecrosis of the Jaw: Case Report. Braz Dent J.

[4] Pichardo SE, Richard van Merkesteyn JP (2013). Bisphosphonate-related osteonecrosis of the jaws: spontaneous or dental origin?. Oral Surg Oral Med Oral Pathol Oral Radiol.

[5] Allen MR, Burr DB (2009). The pathogenesis of bisphosphonate-related osteonecrosis of the jaw: so many hypotheses, so few data. J Oral Maxillofac Surg.

[6] Ruggiero SL, Dodson TB, Fantasia J, Goodday R, Aghaloo T, Mehrotra B (2014). American Association of Oral and Maxillofacial Surgeons position paper on medication-related osteonecrosis of the jaw--2014 update. J Oral Maxillofac Surg.

[7] Yamashita J, McCauley LK (2012). Antiresorptives and osteonecrosis of the jaw. J Evid Based Dent Pract.

[8] Oliveira CC, Brizeno LA, Sousa FB, Mota MR, Alves AP (2016). Osteonecrosis of the jaw induced by receptor activator of nuclear factor-kappa B ligand (Denosumab). Med Oral Patol Oral Cir Bucal.

[9] Otto S, Pautke C (2015). Treatment of medication-related osteonecrosis of the jaw. Medication Related Osteonecrosis of the Jaws: Bisphosphonates, Denosumab and New Agents. Springer-Verlog Berlin Heidelberg.

[10] Yoneda T, Hagino H, Sugimoto T, Ohta H, Takahashi S, Soen S (2017). Antiresorptive agent-related osteonecrosis of the jaw: Position Paper 2017 of the Japanese Allied Committee on Osteonecrosis of the Jaw. J Bone Miner Metab. 2017. J Bone Miner Metab.

[11] Lau AN, Adachi JD (2009). Resolution of osteonecrosis of the jaw after teriparatide [recombinant human PTH-(1-34)] therapy. J Rheumatol.

[12] Martins MA, Martins MD, Lascala CA, Curi MM, Migliorati CA, Tenis CA (2012). Association of laser phototherapy with PRP improves healing of bisphosphonate-related osteonecrosis of the jaws in cancer patients: a preliminary study. Oral Oncol.

[13] Curi MM, Cossolin GS, Koga DH, Zardetto C, Christianini S, Feher O (2011). Bisphosphonate-related osteonecrosis of the jaws–an initial case series report of treatment combining partial bone resection and autologous platelet-rich plasma. J Oral Maxillofac Surg.

[14] Bocanegra-Pérez S, Vicente-Barrero M, Knezevic M, Castellano-Navarro JM, Rodríguez-Bocanegra E, Rodríguez-Millares J (2012). Use of platelet-rich plasma in the treatment of bisphosphonate-related osteonecrosis of the jaw. Int J Oral Maxillofac Surg.

[15] Mozzati M, Gallesio G, Arata V, Pol R, Scoletta M (2012). Platelet-rich therapies in the treatment of intravenous bisphosphonate-related osteonecrosis of the jaw: a report of 32 cases. Oral Oncol.

[16] Dohan Ehrenfest DM, Rasmusson L, Albrektsson T (2009). Classification of platelet concentrates: from pure platelet-rich plasma (P-PRP) to leucocyte- and platelet-rich fibrin (L-PRF). Trends Biotechnol.

[17] Dinca O, Zurac S, Staniceanu F, Bucur MB, Bodnar DC, Vladan C (2014). Clinical and histopathological studies using fibrin-rich 'plasma in the treatment of biphosphonate-related osteonecrosis of the jaw. Rom J Morphol Embryol.

[18] Lesclous P, Grabar S, Najm SA, Carrel JP, Lombardi T, Saffar JL (2014). Relevance of surgical management of patients affected by bisphosphonate-associated osteonecrosis of the jaws. A prospective clinical and radiological study. Clin oral Investig.

[19] Rupel K, Ottaviani G, Gobbo M, Contardo L, Tirelli G, Vescovi P (2014). A systematic review of therapeutical approaches in bisphosphonates-related osteonecrosis of the jaw (BRONJ). Oral Oncol.

